# Calciprotein particle counts associate with vascular remodelling in chronic kidney disease

**DOI:** 10.1093/cvr/cvae164

**Published:** 2024-08-05

**Authors:** Lian Feenstra, Melanie Reijrink, Andreas Pasch, Edward R Smith, Lotte M Visser, Marian Bulthuis, Monique E Lodewijk, Mirjam F Mastik, Marcel J W Greuter, Riemer H J A Slart, Douwe J Mulder, Robert A Pol, Charlotte A te Velde-Keyzer, Guido Krenning, Jan-Luuk Hillebrands, TransplantLines Investigators, V Adelita Ranchor, Antonio W Gomes Neto, Arjan Diepstra, G Bouke Hepkema, C Tji Gan, Caecilia S E Doorenbos, Charlotte A te Velde-Keyzer, Coretta van Leer-Buter, J Daan Touw, Eelko Hak, A M Erik Verschuuren, A J A Frank Bodewes, Frank Klont, Gerard Dijkstra, J Gertrude Nieuwenhuis-Moeke, Hans Blokzijl, G D Henri Leuvenink, Hubert G M Niesters, J Cas Swarte, Jan-Stephan F Sanders, Kevin Damman, L Joost van Pelt, Marco van Londen, Marieke T de Boer, Marion J Siebelink, Marius C van den Heuvel, Michel J Vos, Michiel E Erasmus, Rianne M Douwes, Riemer J H J A Slart, Rinse K Weersma, Robert A Pol, Robert J Porte, Vincent E de Meijer, Willem S Lexmond

**Affiliations:** Department of Pathology and Medical Biology, University of Groningen, University Medical Center Groningen, Hanzeplein 1, 9713 GZ Groningen, The Netherlands; Department of Pathology and Medical Biology, University of Groningen, University Medical Center Groningen, Hanzeplein 1, 9713 GZ Groningen, The Netherlands; Department of Internal Medicine, Division of Vascular Medicine, University of Groningen, University Medical Center Groningen, Groningen, The Netherlands; Calciscon AG, Biel, Switzerland; Institute of Physiology and Pathophysiology, Johannes Kepler University Linz, Linz, Austria; Department of Nephrology, Royal Melbourne Hospital, Parkville, Victoria, Australia; Department of Medicine, University of Melbourne, Parkville, Victoria, Australia; Department of Pathology and Medical Biology, University of Groningen, University Medical Center Groningen, Hanzeplein 1, 9713 GZ Groningen, The Netherlands; Department of Pathology and Medical Biology, University of Groningen, University Medical Center Groningen, Hanzeplein 1, 9713 GZ Groningen, The Netherlands; Department of Pathology and Medical Biology, University of Groningen, University Medical Center Groningen, Hanzeplein 1, 9713 GZ Groningen, The Netherlands; Department of Pathology and Medical Biology, University of Groningen, University Medical Center Groningen, Hanzeplein 1, 9713 GZ Groningen, The Netherlands; Department of Radiology, Medical Imaging Center, University of Groningen, University Medical Center Groningen, Groningen, The Netherlands; Department of Nuclear Medicine and Molecular Imaging, Medical Imaging Center, University of Groningen, University Medical Center Groningen, Groningen, The Netherlands; Department of Biomedical Photonic Imaging, Faculty of Science and Technology, University of Twente, Enschede, The Netherlands; Department of Internal Medicine, Division of Vascular Medicine, University of Groningen, University Medical Center Groningen, Groningen, The Netherlands; Department of Vascular and Transplant Surgery, University of Groningen, University Medical Center Groningen, Groningen, The Netherlands; Department of Internal Medicine, Division of Nephrology, University of Groningen, University Medical Center Groningen, Groningen, The Netherlands; Department of Clinical Pharmacy and Pharmacology, Division Experimental Pharmacology, University of Groningen, University Medical Center Groningen, Groningen, The Netherlands; Department of Pathology and Medical Biology, University of Groningen, University Medical Center Groningen, Hanzeplein 1, 9713 GZ Groningen, The Netherlands

**Keywords:** Chronic kidney disease (CKD), Vascular calcification, Vascular remodelling, Endothelial activation, Calciprotein particles (CPPs), Calcification propensity (crystallization time, T_50_)

## Abstract

**Aims:**

Calciprotein particles (CPPs) are circulating calcium and phosphate nanoparticles associated with the development of vascular calcification (VC) in chronic kidney disease (CKD). Although recent studies have been focusing on associations of CPPs with the presence of VC in CKD, insights in the underlying processes and mechanisms by which CPPs might aggravate VC and vascular dysfunction *in vivo* are currently lacking. Here, we assessed the overall burden of abdominal VC in healthy kidney donors and CKD patients and subsequently performed transcriptome profiling in the vascular tissue obtained from these subjects, linking outcome to CPP counts and calcification propensity.

**Methods and results:**

Calcification scores were quantified in renal arteries, iliac arteries, and abdominal aorta using computed tomography (CT) scans of kidney donors and CKD patients. The vascular tissue was collected from kidney donors (renal artery) and CKD patients (iliac artery), after which bulk RNA sequencing and gene set enrichment analysis (GSEA) were performed on a subset of patients. Calcification propensity (crystallization time, T_50_) was measured using nephelometry and CPP counts with microparticle flow cytometric analysis. Increased calcification scores (based on CT) were found in CKD patients compared to kidney donors. Transcriptome profiling revealed enrichment for processes related to endothelial activation, inflammation, extracellular matrix (ECM) remodelling, and ossification in CKD vascular biopsies compared to kidney donors. Calcification propensity was increased in CKD, as well as CPP counts, with the latter being significantly associated with markers of vascular remodelling.

**Conclusion:**

Our findings reveal that CKD is characterized by systemic VC with increased calcification propensity and CPP counts. Transcriptome profiling showed altered vascular gene expression with enrichment for endothelial activation, inflammation, ECM remodelling, and ossification. Moreover, we demonstrate, for the first time, that vascular remodelling processes are associated with increased circulating CPP counts. Interventions targeting CPPs are promising avenues for alleviating vascular remodelling and VC in CKD.

## Graphical Abstract

**Graphical Abstract:**
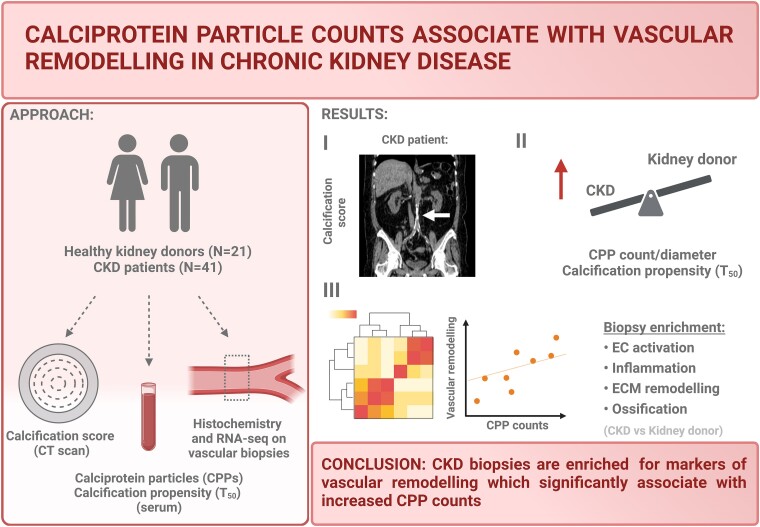

**Time of primary review: 30 days**

## 1. Introduction

Chronic kidney disease (CKD) patients are at an increased risk for the development of cardiovascular diseases (CVD) (e.g. atherosclerosis, medial calcification).^1,2^ As a consequence of reduced renal function, CKD patients exhibit the so-called ‘CKD-related risk factors’, including albuminuria, uremic toxins, volume overload, and high levels of phosphate, which accelerate the development of CVD.^1–3^ High phosphate levels in the circulation are especially of interest in CKD since phosphate is known to bind with the circulating calcium, leading to the formation of *calciprotein particles* (CPPs). CPPs consist of calcium and phosphate mixed with serum proteins like Fetuin-A and albumin.^4,5^ Increased maturation of CPPs (i.e. ripening of primary CPPs (CPP1) to secondary, mature crystalline, CPPs (CPP2)) promotes the calcification propensity, which can be measured as transition time (crystallization time, T_50_), and is used as a biomarker to estimate the cardiovascular risk in CKD.^6–8^

The development of vascular calcifications (VC) can be divided into two different forms based on anatomical location: intimal and medial calcification.^2^ Intimal calcification generally results from dyslipidaemia and occurs mainly in the intima layer (i.e. the subendothelial space) of the vascular wall. Intimal calcification is characterized by activated endothelial cells (ECs) and fibrous plaque formation.^9,10^ Interestingly, it has been shown that *ex vivo* perfusion of atherosclerotic vessels with labelled CPPs results in lesional uptake of the particles in the vessel.^11^ In contrast to intimal calcification, medial calcification (i.e. Mönckeberg's sclerosis) frequently develops in the absence of lipid deposition. Medial calcification is known to involve the vascular smooth muscle cells (VSMCs) primarily, where VSMCs change from a contractile to an osteochondrogenic phenotype.^10,12^ Additionally, VSMCs have been shown to take up CPPs from the environment (*in vitro*), which most likely promotes the process of medial VC.^13,14^ Although intimal and medial calcifications develop via two distinct pathways, both forms are not mutually exclusive and frequently coexist in CKD.^2,15^

Until now, numerous studies have investigated associations between increased calcification propensity (reduced T_50_) and the development of vascular diseases in CKD. For example, in a CKD cohort (stage 2-4), lower T_50_ values (i.e. accelerated CPP crystallization) were measured in patients with a higher coronary artery calcium (CAC) score.^16^ In a cohort of renal transplant recipients, reduced serum T_50_ has been associated with all-cause mortality and increased risk for graft failure.^17^ Similarly, in a patient population receiving haemodialysis, calcification propensity is independently associated with all-cause mortality, myocardial infarction, and peripheral vascular events.^18^ Moreover, in a CKD cohort (stage 3-4) an association was found between progressive aortic stiffening and serum T_50_ values.^19^ While most of these studies focus on calcification propensity as measure for CPP2 formation and the associations hereof with the development of CVD in CKD, *in vivo* data on the molecular and process-related signature of VC and vascular dysfunction in CKD are largely lacking.

In the current study, we aimed to unravel the underlying biological processes of VC and vascular dysfunction in CKD and associate this with pro-calcifying determinants, including calcification propensity and CPP counts. Hereto, we analysed a unique series of vascular tissue obtained from healthy kidney donors and CKD patients during kidney procurement and transplantation. Transcriptome profiling on the vascular biopsies allowed for associations of vascular gene expression patterns to circulating CPP counts and serum calcification propensity.

## 2. Material and methods

### 2.1 Study design

This single centre, cross-sectional study was conducted as a sub-study of the TransplantLines Biobank and cohort study, which is a large longitudinal cohort study investigating several outcomes after organ transplantation of patients in the University Medical Center Groningen (UMCG).^20^ The study was performed in compliance with the principles of the Declaration of Helsinki. All subjects [chronic kidney disease patients (CKD) (*N* = 41), healthy kidney donors (*N* = 21)] provided written informed consent. The protocol was reviewed and approved by the Institutional Review Board of the UMCG (number 2014-077). Patients were included between November 2017 and November 2020.

### 2.2 Vascular tissue collection

To assess alterations in vascular tissue morphology and gene expression levels, vascular biopsies were procured from CKD and healthy living kidney donors during organ transplantation. External iliac artery (EIA) punch biopsies were obtained from CKD patients before anastomosis of the graft's renal artery to the recipient's EIA. From the healthy living kidney donors, a small ring (approximately 2 mm) of the renal artery was collected and utilized as a control for healthy vascular tissue. Vascular tissue biopsies were divided in two parts, that is, either formalin-fixed or snap-frozen for morphological and gene expression analysis, respectively. Of note, CKD patients were matched with either healthy living kidney donors or multi-organ donors (MOD). In this study, healthy living kidney donors were included as controls for analyses, explaining differences in biopsy numbers (*N*) between donors (*N* = 21) and CKD patients (*N* = 41). For RNA-sequencing analysis (see 2.7 below), external iliac artery biopsies were obtained from multi-organ donors (MOD) (*N* = 6).

### 2.3 Clinical and laboratory assessments

Serum and plasma samples from CKD patients and healthy kidney donors were collected at the day prior to organ transplantation. Haemoglobin A1c (HbA_1c_), lipid profile, C-reactive protein (CRP), leucocyte count, calcium, phosphate, and alanine aminotransferases were measured according to standard routine procedures. eGFR was calculated using the CKD-EPI formula (chronic kidney disease epidemiology collaboration).^21^ Demographic parameters like age, gender, BMI, and history of dialysis treatment were collected as well. For this study patients with type 1 diabetes mellitus (T1DM) were excluded.

### 2.4 Computed tomography imaging

Computed tomography (CT) scans of healthy kidney donors and CKD patients were analysed with Aquarius iNtuition viewer P4 (TeraRecon, USA) according to the Rominger calcification score.^22^ Calcification scores were classified as follows: score 0 (no visible calcification), score 1 (<10% of the arterial wall is calcified), score 2 (10–25% of the arterial wall is calcified), score 3 (25–50% of the arterial wall is calcified), and score 4 (>50% of the arterial wall is calcified). Scores were based on the CT slice demonstrating most calcification. The abdominal aorta, both renal arteries, both common iliac arteries, and the EIAs on both sides (left and right) were scored separately. The renal arteries were analysed on sagittal slices, and the abdominal aorta, common iliac arteries, and EIAs were analysed on transversal slices. The total abdominal artery calcification score was computed by summing all seven evaluated areas, with a maximum score of 28. The renal artery calcification score was calculated as the combined score for the left and right renal arteries. The iliac calcification score was the sum of the common iliac arteries and EIAs (left and right). For this analysis, a subpopulation of the total clinical cohort was used based on the availability of scans.

### 2.5 Histological staining

The vascular tissue was formalin-fixed, paraffin-embedded, and cut in 3 µm tissue sections. Presence of microcalcifications was evaluated using Alizarin red and von Kossa staining. Tissue sections were first deparaffinized in xylene and rehydrated in graded series of EtOH. For the Alizarin red staining, tissue sections were incubated with a 2% Alizarin red (pH = 4.2) solution for 5 min room temperature (RT). Thereafter, sections were washed with an acetone–xylene solution (1:1) and dehydrated with 100% xylene. For the von Kossa staining, sections were incubated with 1% silver nitrate for 1 h RT in daylight, followed by 3% sodium thiosulfate exposure for 5 min RT. Sections were counterstained with Nuclear Fast Red for 3 min RT. Both the Alizarin red and von Kossa stained-sections were mounted with Tissue-Tek Film (Sakura Finetek, USA). Slides were scanned with the NanoZoomer S360 digital slide scanner (Hamamatsu, Japan). Scans were analysed using the ImageScope software package (V.12.4.3.5008, Aperio, Leica Biosystems Imaging, USA). Scans were analysed semi-quantitatively based on the following criteria: score 0 (no calcification), score 1 (traces of microcalcification), score 2 (mild microcalcification), score 3 (moderate microcalcification), and score 4 (severe microcalcification). For this analysis, a subpopulation of the total clinical cohort was used based on the availability of tissue.

### 2.6 Quantitative real-time polymerase chain reaction

To measure alterations in VSMC dedifferentiation and calcification marker gene expression, quantitative real-time polymerase chain reaction (qRT-PCR) analyses were performed. Hereto, snap-frozen vascular biopsies were sectioned and lysed in QIAzol lysis reagent. Subsequently, RNA was isolated using the RNeasy Mini Kit (#74106, Qiagen, The Netherlands). cDNA was synthesized using SuperScript II Reverse Transcriptase (#18064014, ThermoFisher Scientific, USA). For the qRT-PCR, 1 ng of cDNA was combined with TaqMan assays for the following genes: Actin Alpha 2 (ACTA2), Myosin Heavy Chain 11 (MYH11), Transgelin (TAGLN), Alkaline Phosphatase (ALPL), Integrin Binding Sialoprotein (IBSP), Matrix Gla Protein (MGP), Muscle Segment Homeobox 2 (MSX2), Runt-related Transcription Factor 2 (RUNX2), and SRY-Box Transcription Factor 9 (SOX9). For input reference β-actin was used. Primer sequences, assay IDs, and amplicon lengths can be found in Supplementary material online, *Table S1*. Cp values below the detection limit were set at a value of 40. Relative expression was calculated as 2^^−deltaCp^ and shown in each graph. For this analysis, a subpopulation of the total clinical cohort was used based on the availability of tissue.

### 2.7 Bulk RNA-sequencing and gene set enrichment analysis

To assess alterations in vascular gene expression patterns, additional bulk RNA-sequencing was performed on vascular biopsies. Vascular biopsies from CKD patients (*N* = 10), living kidney donors (*N* = 10), and MOD (*N* = 6) selected for RNAseq were matched for age and gender. Additionally, in the CKD group, biopsies were further selected based on plasma calcium and phosphate levels, arterial calcification score, and the presence of patients on haemodialysis. RNA sequencing was performed by GenomeScan (Leiden, The Netherlands). Brief, RNA (40 ng) was depleted of ribosomal RNA using the NEBNext rRNA depletion kit (#E6310, New England Biolabs, USA). Sample libraries were prepared with the NEBNext Ultra II Directional RNA Library Prep Kit for Illumina (#7760, New England Biolabs, USA). Paired-end sequencing was executed on a NovaSeq 6000 System (Illumina, USA) using the Illumina data analysis pipeline RTA3.4.4 and Bcl2fastq v2.20 (Illumina, USA).

Next, Galaxy environment at usegalaxy.org was used for sequencing alignment.^23^ First, adapter sequences and the leading 20 bases were trimmed using Trimmomatic (v.0.38.0) program. The obtained sequences were aligned to the human reference genome (build hg38) using HiSAT2 (v. 2.1.0) and fragments counted by HTSeq-count (v. 0.9.1) in union mode using the human reference transcriptome (version 104) as reference [available through Ensembl (Homo_sapiens.GRCh38.104.chr.gtf)] and used to determine differentially expressed genes by the DESeq2 (v.2.11.40.6) algorithm with standard parameters. Samples that raised sequencing errors, had insufficient sequencing depth, or raised critical quality control issues (FastQC, v.0.11.8) were flagged as potential outlier and excluded from the study after their discordant behaviour was confirmed by principal component analysis using robust Mahalanobis distance.^24^ To be able to control for the potential confounding effects of artery type on differential gene expression in RNAseq analysis, iliac artery biopsies obtained from deceased MOD were also included for comparison. Outlier elimination resulted in a final sample set of 18 samples (*n* = 6 CKD iliac artery biopsies, *n* = 6 living kidney donor renal artery biopsies, *n* = 6 MOD iliac artery biopsies). Baseline characteristics of the arterial biopsy donors used for RNAseq are shown in Supplementary material online, *Table S2*. For gene expression analysis, genes that were not expressed (raw counts = 0) or had low abundance (<10 raw counts/sample in over half of the samples) were filtered out, and multiple testing correction was performed by the Benjamini–Hochberg procedure to control the false discovery rate (FDR). DESeq2-normalized sequence counts were used for the gene set enrichment analysis (GSEA, v. 4.1.0)^25^ using the curated C5 collection of Cellular Biological Processes within the MSigDB database (v.7.4).^26^ Enrichment analysis for the significantly differentially expressed genes associated with CPP1 or CPP2 count (*P*-value < 0.05) was performed with Enrichr–Reactome 2022 database.^27–29^

### 2.8 Calcification propensity (T_50_) assessment

Serum T_50_ measurements were carried out using a slightly modified version of the original Pasch *et al.*^8^ procedure. To summarize, 40 µL of serum samples was placed into clear 96-well plates, and the serum was then gradually mixed with 35 µL of calcium solution and 25 µL of phosphate solution. A Freedom Evo 100 liquid handling robot (Tecan, Männedorf, Switzerland) was used for every pipetting phase. Every liquid was added, and then an orbital shaker was used to stir the mixture. After that, the plate was sealed with an adhesive film and placed within a temperature-controlled nephelometer (NEPHELOstar PLUS, BMG Labtech, Ortenberg, Germany) set to 37°C. For 10 h, a kinetic measurement was taken in each well every 3 min. The in-house T_50_ analysis software of Calciscon analysed the kinetic raw data for T_50_ determination. The average intra-assay coefficient of variations (CV) for test performances was 2.2%, whereas the average inter-assay CV was 3.4%. The T_50_ test reference range, as established in sera from 253 healthy Swiss blood volunteers, was 270–470 min. Lastly, samples positive for gross lipidaemia or haemolysis were excluded. Sample sizes were based on the availability of serum and represented a sub-population of the total clinical cohort.

### 2.9 Calciprotein particle quantification

CPP measurements (CPP1 and CPP2 counts) were performed according to our previously published methods,^30,31^ with minor modifications. All solutions were twice 0.22 µm-filtered prior to use. Bovine fetuin-A was obtained from Sigma (#F3004, Sigma-Aldrich, USA) and the monomer purified by size exclusion chromatography, as described previously.^32^ Frozen serum samples were thawed in a water bath at 37°C with gentle agitation. Subsequently, 5 µL portions were mixed with 40 µL HEPES-buffered Dulbecco's modified Eagle's medium (DMEM) (50 mM HEPES, no phenol red, pH 7.45) and 5 µL staining solution containing Alexa Fluor 647-conjugated risedronate (5 µM, #BV500101, BioVinc, USA), FITC-conjugated lactadherin (1.5 µg/mL, #BLAC-FITC, Haematologic Technologies Inc., USA), and mFluor violet 450-labelled bovine fetuin-A (2 µg/mL, prepared in-house using ReadiLink™ Rapid mFluor™ Violet 450 Labelling Kit, #1100, AAT Bioquest, USA). After 120 min incubation in the dark with gentle mixing, samples were diluted to 500 µL in HEPES-buffered DMEM. Measurements were acquired in triplicate using a calibrated Apogee A50/Microflow Cytometer equipped with 50 mW 405, 488, and 638 nm lasers (parameter settings: sheath pressure 150 mbar, four flush cycles). Flow rate (3 µL/min) and measurement times (120 s or until data storage buffer was full—5 000 000 events) were held constant for all samples. Fluorescence threshold triggering was used to detect fetuin-A-stained particles, and a negative gating strategy was used to exclude membrane-delimited particles, which stain positive for FITC-conjugated lactadherin. CPPs were gated as fetuin-A^+^ lactadherin^−^ events. Amorphous calcium phosphate-containing (CPP1) and crystalline hydroxyapatite-containing (CPP2) populations were gated based on their differential affinity for risedronate: risedronate^LO^ and risedronate^HI^, respectively. Samples positive for gross lipidaemia or haemolysis were excluded. Sample sizes were based on the availability of serum and represent a sub-population of the total clinical cohort.

### 2.10 Statistical analysis

Statistical analyses of experimental data were performed with GraphPad Prism 10.0.3 (GraphPad Software Inc., USA). Differences between two groups were assessed with a Mann–Whitney U test. Statistical analyses on clinical demographics were performed with IBM Statistical Package for Social Sciences (SPSS) version 23. Demographical data are presented as mean ± standard deviation (SD) and tested with unpaired *t*-test, or median ± interquartile range (IQR) and tested with Mann–Whitney U test (continuous data). Categorical data were tested with the χ^2^ test. *P* < 0.05 was considered statistically significant. Graphs were generated with GraphPad Prism and figures created with Adobe Illustrator 27.7 (Adobe Inc., USA).

## 3. Results

### 3.1 Study population characteristics

Demographic and clinical characteristics of all chronic kidney disease patients (CKD, *N* = 41) and healthy kidney donors (*N* = 21) are summarized in *Table 1*. In total, 57.1% of the CKD patients vs. 47.6% of the healthy kidney donors were male. Age distribution was similar between both groups (55 ± 13 years vs. 50 ± 11 years in CKD and kidney donors, respectively). The estimated glomerular filtration rate (eGFR) was lower in CKD patients compared to kidney donors [7.5 (6.0–10) vs. 87 (80–101), respectively]. Among CKD patients, 47.6% were on dialysis prior to transplantation, and 35.7% were diagnosed with type 2 diabetes. C-reactive protein was significantly higher in CKD patients [2.5 (1.1–6.0) vs. 1.4 (0.5–3.3) in CKD and kidney donors, respectively, *P* < 0.05), while alanine aminotransferase was significantly lower in CKD patients compared to kidney donors [16 (13–23) vs. 21 (18–30) in CKD and kidney donors respectively, *P* < 0.05]. Furthermore, CKD patients exhibited significantly higher serum phosphate levels compared to kidney donors [1.5 (1.3–1.9) vs. 0.9 (0.9–1.1) in CKD and kidney donors, respectively, *P* < 0.001]. Other measured parameters were comparable between groups (*Table 1*).

**Table 1:** Baseline characteristics of the healthy kidney donors and CKD patients

	Chronic kidney disease patients (CKD, *N* = 41)	Healthy kidney donors (*N* = 21)	*P*-value
Male gender (%)	57.1	47.6	0.327
Age (years)	55 ± 13	50 ± 11	0.124
Body mass index (kg/m^2^)	27 ± 4.0	27 ± 2.7	0.474
Dialysis (%)	47.6	0	<0.001
Estimated glomerular filtration rate (mL/min/1.73 m^2^)	7.5 [6.0–10]	87 [80–101]	<0.001
Type 2 diabetes (%)	35.7	0	<0.001
Diabetes duration (years)	13 [8.0–20]^b^		
HbA1_C_ (%)	5.4 [5.0–6.0]	5.4 [5.2–5.6]	0.770
Total cholesterol (mmol/L)	4.7 ± 1.3	4.8 ± 1.0	0.654
C-reactive protein (mg/L)	2.5 [1.1–6.0]	1.4 [0.5–3.3]	0.046
Leucocyte count (10^9^/L)	7.0 ± 2.0	7.0 ± 2.4*	0.998
Calcium (mmol/L)	2.3 ± 0.2	2.4 ± 0.09	0.735
Phosphate (mmol/L)	1.5 [1.3–1.9]	0.9 [0.9–1.1]	<0.001
Alanine aminotransferase (U/L)	16 [13–23]	21 [18–30]	0.010

Data are presented as mean ± SD (when normally distributed) or as median with [IQR] (when not normally distributed).

^*^ *N* = 20.

^b^N = 15, *P*-value based on unpaired *t*-test when normally distributed or Mann–Whitney U test when not normally distributed or χ^2^ test for categorical data (gender, dialysis, and type 2 diabetes mellitus).

### 3.2 CKD patients present with increased CT-assessed (macro)calcifications

**Figure 1:**
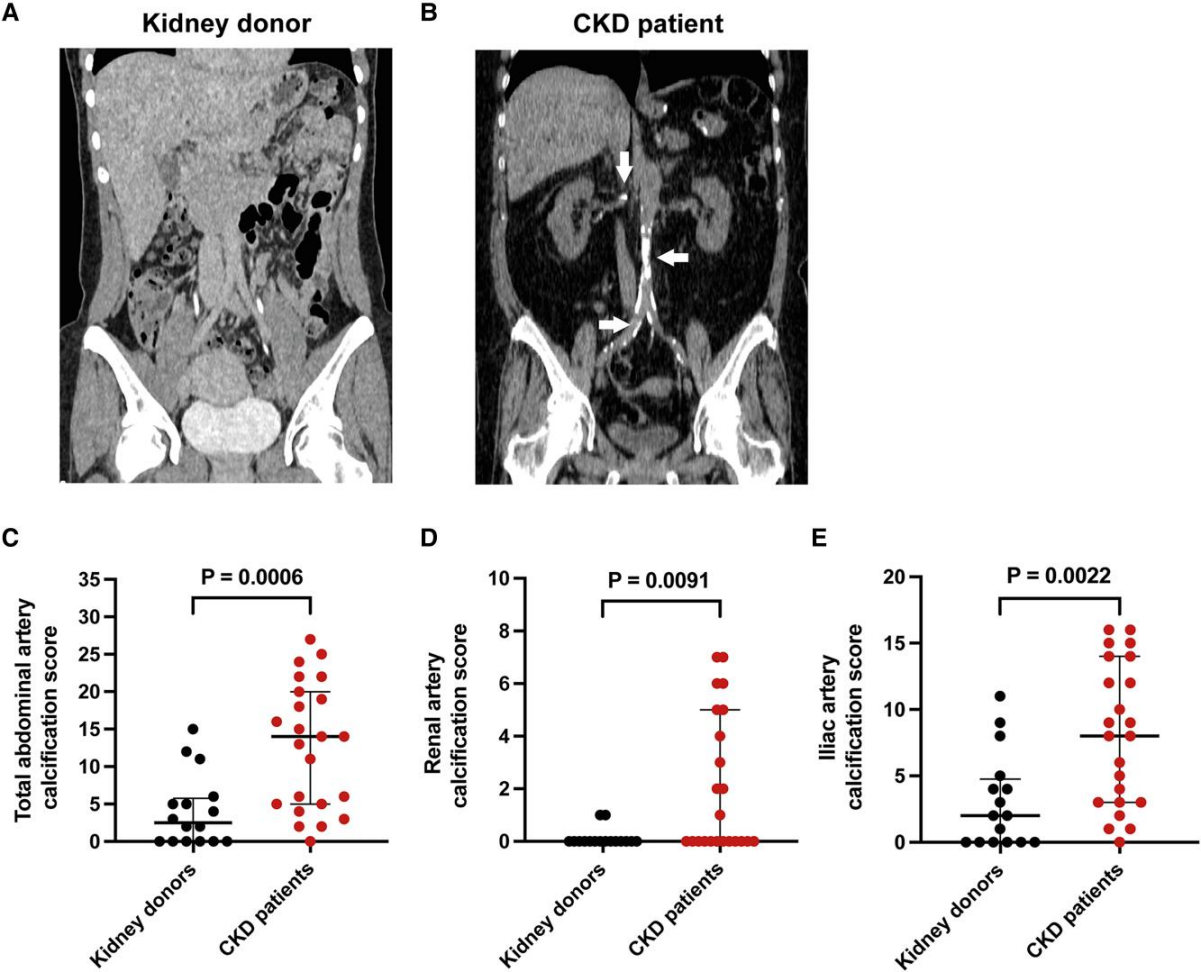
Computed tomography-assessed (macro)calcifications in healthy kidney donors and CKD patients. (*A*, *B*) Representative image of the computed tomography (CT) scan of (*A*) healthy kidney donors or (*B*) CKD patients. Arrows indicate spots of (macro)calcification. (*C*–*E*) Quantification of the CT-assessed (macro)calcifications resulted in a total abdominal artery calcification score (sum of all seven abdominal arteries) (*C*), renal artery calcification score (sum left and right renal artery) (*D*), and iliac artery calcification score (sum external and common iliac arteries, left and right) (*E*). Graphs show medians with interquartile ranges and individual data points (kidney donors: *n* = 16; CKD patients: *n* = 23). *P* < 0.05 was considered statistically significant and tested using a Mann–Whitney U test.

To assess the presence of (macro)calcifications in the vasculature of kidney donors and CKD patients, the total abdominal artery calcification score was assessed using computed tomography (CT). Additionally, we quantified the renal and iliac artery calcification scores as well since RNA sequencing was performed on these arteries. The total abdominal artery calcification score was available for 16 kidney donors and 23 CKD patients. Representative visualisations of the CT-assessed vascular (macro)calcifications in kidney donors and CKD patients are demonstrated in *Figure 1A* and *B*, respectively. When comparing the total abdominal artery calcification score (sum all seven abdominal arteries, *Figure 1C*), as well as the renal artery (sum left and right renal artery, *Figure 1D*) and iliac artery (sum common and external iliac arteries, left and right, *Figure 1E*) calcification scores, significantly increased calcification scores were observed in the CKD patients compared to kidney donors. These findings suggest that (macro)calcifications, as indicated by the total abdominal artery calcification score, are more prevalent [22 out of 23 (96%) CKD patients vs. 10 out of 16 (63%) kidney donors, *Figure 1C*] and more severe (i.e. higher score) in CKD patients compared to kidney donors.

### 3.3 Histological analysis reveals presence of microcalcification in vascular tissue of CKD patients

**Figure 2:**
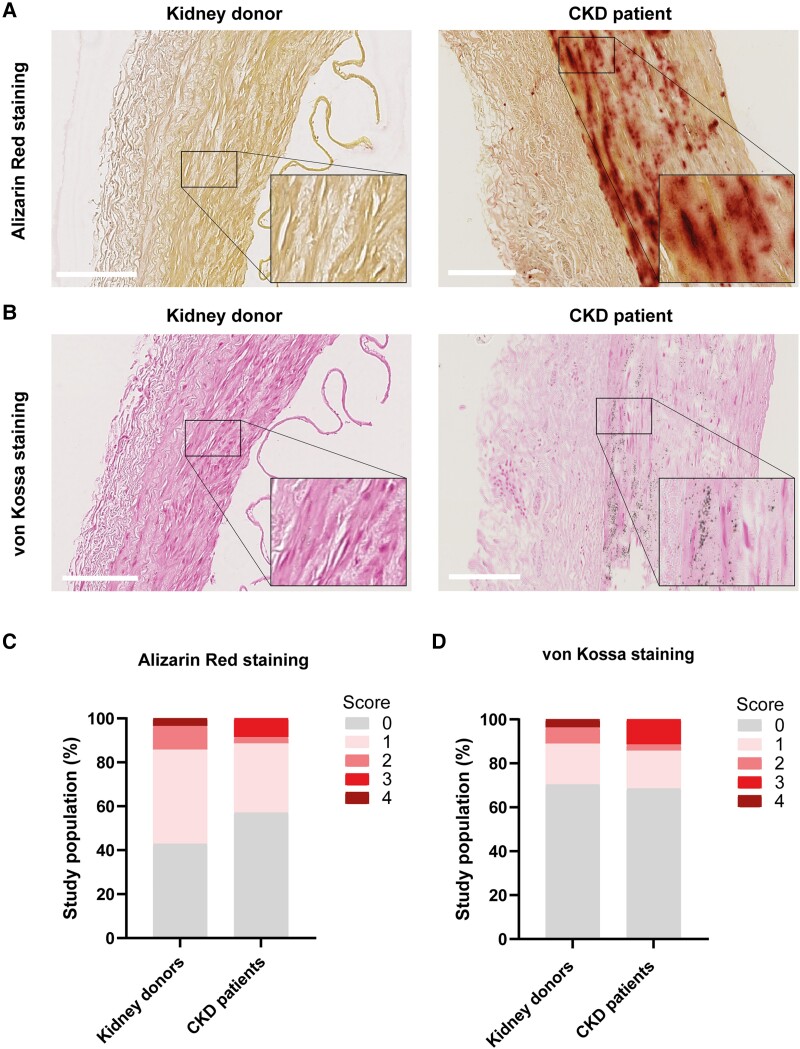
Histological analysis of microcalcifications in healthy kidney donors and CKD patients. (*A*, *B*) Representative image of Alizarin red staining (*A*) and von Kossa staining (*B*) on vascular biopsies of healthy kidney donors (renal artery) and CKD patients (iliac artery) to assess microcalcifications. Calcification is visible as red-coloured calcium-rich deposits (Alizarin red staining) or black-coloured calcium-phosphate-rich deposits (von Kossa staining). Scale bar represents 200 μm. (*C*, *D*) Quantification of Alizarin red (*C*) or von Kossa (*D*) staining on the vascular tissue of healthy kidney donors and CKD patients (kidney donors: *n* = 28; CKD patients: *n* = 35). Data are shown in stacked bar graphs: score 0 (no calcification), score 1 (traces of microcalcification), score 2 (mild microcalcification), score 3 (moderate microcalcification), and score 4 (severe microcalcification).

Next to the CT-based macrocalcifications, we also assessed the presence of microcalcifications at the tissue level in the vascular biopsies using Alizarin red and von Kossa staining (*Figure 2A* and *B*). Generally, the histology-confirmed presence of microcalcifications was limited in both CKD and kidney donors. When microcalcifications were present, calcified areas were predominantly observed in the media layer of the vessel wall (*Figure 2A* and *B*). Semi-quantitative analysis revealed only a slightly increased media calcification severity in CKD patients compared to kidney donors (score 3, *Figure 2C* and *D*) based on both Alizarin red and von Kossa. Clear differences between CKD recipients and kidney donors for the presence of microcalcifications at the histological level were absent and did not mirror the findings obtained with CT.

### 3.4 Similar expression of classical VSMC dedifferentiation and calcification markers in kidney donors and CKD vascular biopsies

**Figure 3:**
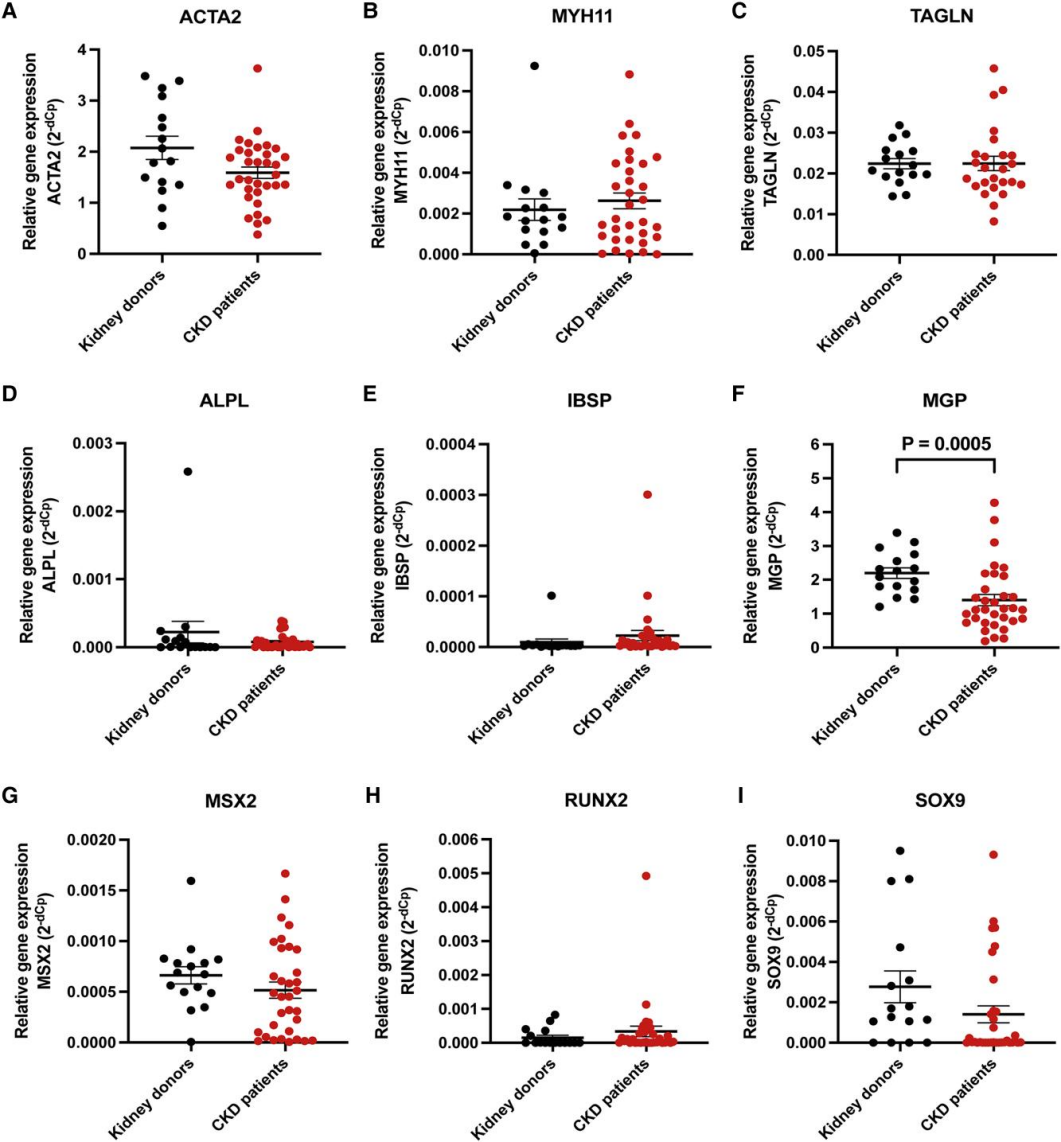
VSMC dedifferentiation and calcification marker gene expression assessed with qRT-PCR. (*A*–*C*) mRNA expression of VSMC osteochondrogenic dedifferentiation markers ACTA2 (*A*), MYH11 (*B*), and Transgelin (TAGLN) (*C*). (*D*–*I*) mRNA expression of VSMC calcification markers ALPL (*D*), IBSP (*E*), MGP (*F*), MSX2 (*G*), RUNX2 (*H*), and SOX9 (*I*). Data are shown as relative expression (2^−dCp^) (means ± SEM) and individual data points (*n* = 15–33, variable since not all genes were analysed on all samples). Significance is indicated *P* < 0.05 and tested with a Mann–Whitney U test.

To assess VSMC phenotype switching from a contractile to osteoblast-like phenotype (i.e. a process frequently observed during VC^33^) in CKD, gene expression analysis of various VSMC dedifferentiation and calcification markers in vascular biopsies from both kidney donors and CKD patients was performed. No differences were observed in the expression of VSMC dedifferentiation markers ACTA2, MYH11, or Transgelin (TAGLN) (*Figure 3A–C*). Furthermore, the expressions of active calcification-associated VSMC markers ALPL, IBSP, MSX2, RUNX2, and SOX9 were comparable between vascular biopsies obtained from kidney donors and CKD patients (*Figure 3D*, *E* and *G–I*). Among the markers analysed, only the expression of the anti-calcification marker MGP was significantly lower in CKD compared to kidney donors (*Figure 3F*). It is noteworthy that, in general, gene expression levels of most calcification markers were extremely low. Taken together, based on the markers tested, the gene expression analysis did not reveal a significant activation of dedifferentiation or pro-calcification pathways within the vascular biopsies from CKD patients. These findings align with the histological analyses described above.

### 3.5 Transcriptome analysis reveals endothelial activation, inflammation, ECM remodelling, and ossification enriched in vascular tissue of CKD patients compared to healthy kidney donors

**Figure 4:**
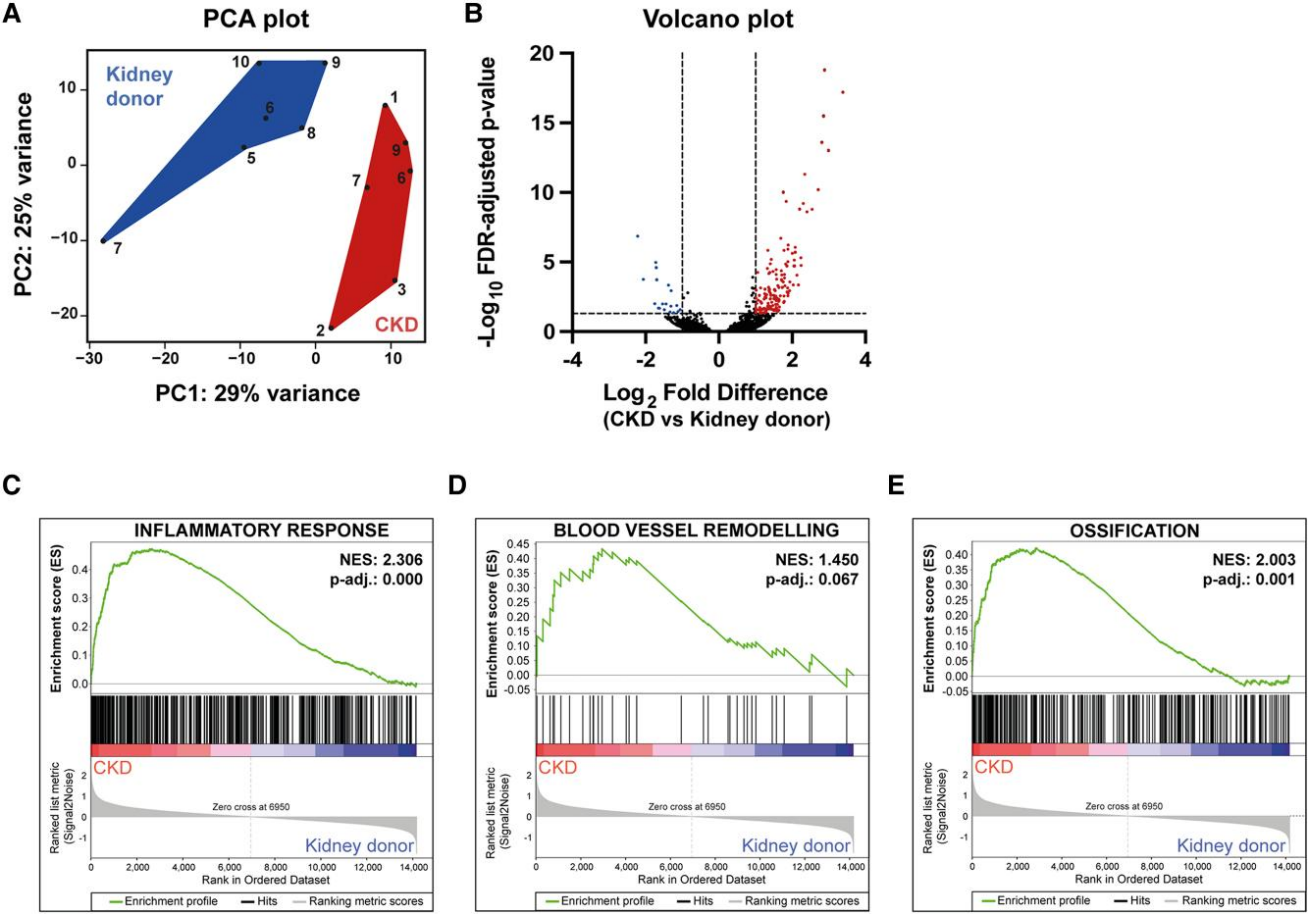
Bulk RNA sequencing on vascular biopsies of healthy kidney donors and CKD patients. (*A*) PCA plot showing the distribution of the data of healthy kidney donors and chronic kidney disease (CKD) patients. (*B*) Volcano plot showing differentially expressed genes using Log_2_ fold difference >1 and an FDR-adjusted *P*-value < 0.05. Significantly increased genes in CKD are indicated in red (*n* = 147), while significantly decreased genes in CKD are indicated in blue (*n* = 19). Plot shows the ratio CKD vs. kidney donors. (*C*, *D*) Enrichment plots showing GO term inflammatory response (*C*), blood vessel remodelling (*D*), and ossification (*E*). Normalized enrichment score and FDR-adjusted *P*-value are shown. Black vertical bars indicate all individual hits for the GO term. The enrichment profile is indicated with a green line. Data included healthy kidney donors (*n* = 6) and CKD patients (*n* = 6).

Since no substantial differences were observed in the expression of the initially tested marker genes for dedifferentiation and calcification, additional transcriptome analysis was conducted, combining RNA sequencing with gene set enrichment analysis, to assess process alterations in the mRNA expression beyond the obvious candidate genes. Principal component analysis (PCA) illustrated two distinct sample populations, with kidney donor samples clustering separately from CKD patient samples (*Figure 4A*). PCA revealed a noticeable differential gene expression between the vascular tissues derived from kidney donors and CKD patients. RNA sequencing confirmed the absence of differential expression in the tested marker genes for dedifferentiation and calcification, thus mirroring the gene expression analysis (see Supplementary material online, *Table S3*). Using an FDR adjusted *P*-value < 0.05 and Log_2_ fold difference of >1, 19 genes were significantly downregulated (indicated in blue), and 147 genes were significantly upregulated (indicated in red) in CKD vascular biopsies compared to the healthy kidney donors (*Figure 4B*). A complete list of differentially expressed genes can be found in Supplementary material online, *Table S4*. Gene enrichment analysis showed significant alterations in cellular biological processes, including ‘endothelial cell barrier’, ‘nitric oxide homeostasis’, ‘leucocyte adhesion to vascular endothelial cell’ (included in the ‘endothelial cell activation’ category), and ‘replicative senescence’ (*Table 2*). Additionally, processes, such as ‘smooth muscle cell differentiation, proliferation and migration’ and ‘osteoblast differentiation’, were significantly enriched in the CKD patient-derived biopsies. Within the gene enrichment analysis for biological processes, enrichment within ‘inflammatory response’, ‘cytokine production in inflammatory response’, ‘macrophage activation’, ‘cell redox homeostasis’, vascular remodelling-related processes, such as ‘extracellular matrix assembly/disassembly’, ‘collagen catabolic process’, and the GO term ‘ossification’ were observed (*Table 3*). Enrichment plots are shown for GO terms ‘inflammatory response’, ‘blood vessel remodelling’, and ‘ossification’ (*Figure 4C–E*).

**Table 2:** Gene set enrichment for cellular biological processes in vascular biopsies of CKD patients (iliac artery) vs. healthy kidney donors (renal artery)

	GO term	NES	*P*-value	FDR
**Endothelial cell activation**				
Endothelial cell activation	GO:0042118	1.079	0.331	0.364
Endothelial cell barrier	GO:0061028	1.863	0.002	0.004
Nitric oxide homeostasis	GO:0033484	1.568	0.014	0.035
Leukocyte adhesion to vascular endothelial cell	GO:0061756	1.890	0.000	0.003
**Smooth muscle cell differentiation**				
Smooth muscle cell differentiation	GO:0051145	2.201	0.000	0.000
Smooth muscle cell proliferation	GO:0048659	1.627	0.016	0.025
Smooth muscle cell migration	GO:0014909	1.687	0.010	0.016
Smooth muscle contraction	GO:0006939	1.312	0.143	0.128
Smooth muscle relaxation	GO:0044557	1.415	0.099	0.079
**Osteochondrogenic dedifferentiation**				
Osteoblast development	GO:0002076	1.252	0.207	0.162
Osteoblast differentiation	GO:0001649	1.495	0.057	0.048
Chondrocyte development	GO:0002063	1.229	0.217	0.180
Chondrocyte differentiation	GO:0002062	0.799	0.723	0.827
**Cellular senescence**				
Cellular senescence	GO:0090398	1.067	0.336	0.366
Replicative senescence	GO:0090399	1.502	0.019	0.048
Stress-induced premature senescence	GO:0090400	1.089	0.308	0.359
DNA damage				
Double-strand break repair	GO:0006302	1.370	0.060	0.100

FDR, false discovery rate; GSEA, gene set enrichment analysis; NES, normalized enrichment score.

**Table 3:** Gene set enrichment for biological processes in vascular biopsies of CKD patients (iliac artery) vs. healthy kidney donors (renal artery)

	GO term	NES	*P*-value	FDR
**Inflammatory signalling**				
Inflammatory response	GO:0006954	2.306	0.000	0.000
Acute inflammatory response	GO:0002526	1.534	0.001	0.042
Chronic inflammatory response	GO:0002544	1.539	0.011	0.043
Cytokine production in inflammatory response	GO:0002534	1.806	0.000	0.006
Lymphocyte activation	GO:0046649	1.360	0.046	0.103
Macrophage activation	GO:0042116	1.520	0.010	0.048
Leukocyte migration in inflammatory response	GO:0002523	1.406	0.029	0.082
**Oxidative stress**				
Cell redox homeostasis	GO:0045454	1.597	0.017	0.029
Cellular oxidant detoxification	GO:0098869	1.076	0.337	0.361
**Vascular remodelling**				
Blood vessel remodelling	GO:0001974	1.450	0.051	0.067
Extracellular matrix assembly	GO:0085029	1.908	0.002	0.003
Extracellular matrix disassembly	GO:0022617	1.962	0.000	0.002
Collagen biosynthetic process	GO:0032964	1.298	0.124	0.135
Collagen catabolic process	GO:0030574	2.035	0.000	0.001
**Vascular calcification**				
Biomineralization	GO:0110148	1.339	0.148	0.113
Ossification	GO:0001503	2.003	0.000	0.001

FDR, false discovery rate; GSEA, gene set enrichment analysis; NES, normalized enrichment score.

To assure that the observed differences did not originate from the gene expression signature from different vascular beds, we analysed the differential gene expression between the renal artery from CKD patients and deceased multi-organ donors (MOD). The differential gene expression between CKD and MOD correlated well with the differential gene expression between CKD and KD (r = 0.52, *P* < 0.001), albeit some differences in gene expression (see Supplementary material online, *Figure S1*). When comparing Log_2_FD of ‘CKD vs. MOD’ with ‘CKD vs. KD’, the differential expression of 15 genes (0.08%) was higher, while that of 112 genes (0.62%) was lower in the MOD external iliac arteries compared with the KD renal arteries (see Supplementary material online, *Figure S1*). In direct comparison of the KD renal arteries with MOD iliac arteries (all non-CKD), increased expression of 44 (0.24%) genes and decreased expression of 42 (0.23%) genes were observed in the KD renal arteries (see Supplementary material online, *Table S5*). Nonetheless, the highly overlapping gene expression profiles in both types of control donor vasculatures reinforces the concept that the artery wall in CKD displays a CKD-dependent mRNA expression profile.

### 3.6 Calcification propensity and CPP counts are increased in CKD patients

**Figure 5:**
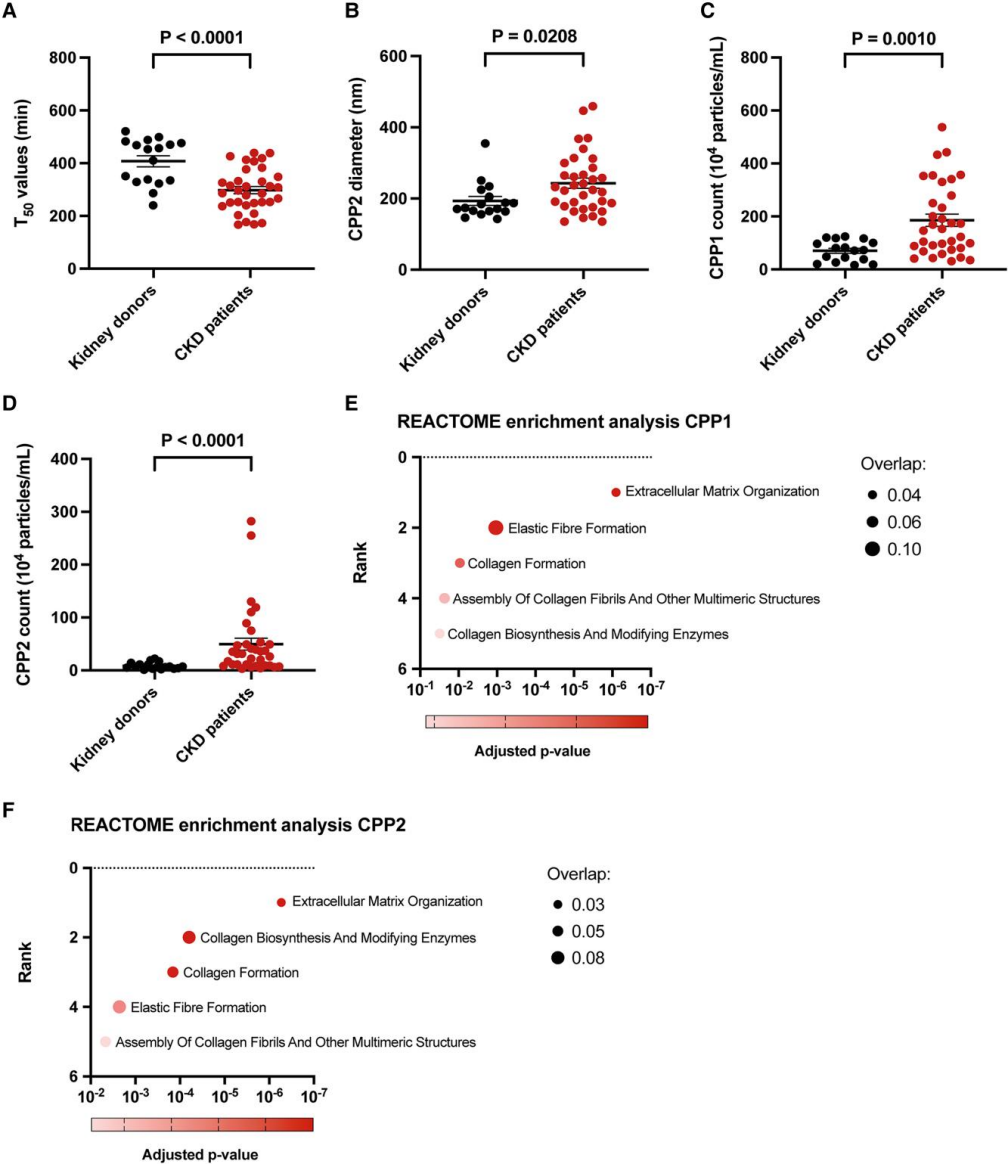
Serum calcification propensity, CPP radius, and CPP count in healthy kidney donors and CKD patients. (*A*) Serum calcification propensity measured as T_50_ values and expressed in minutes (min). (*B*) CPP2 diameter expressed in nanometer (nm). (*C*) Serum CPP1 count expressed as 10^4^ particles per millilitre serum (mL). (*D*) Serum CPP2 count expressed as 10^4^ particles per mL serum. All parameters measured in serum of healthy kidney donors (*n* = 17) and CKD patients (*n* = 34). Data show means ± SEM and individual data points. Significance is indicated *P* < 0.05 and tested with a Mann–Whitney U test. (*E*) Reactome2022 enrichment analysis for differently expressed RNA sequencing genes, which were significantly associated with CPP1 counts (67 genes, *n* = 6 healthy kidney donors and *n* = 6 CKD patients). (*F*) Reactome2022 enrichment analysis for differently expressed RNA-sequencing genes, which were significantly associated with CPP2 counts (51 genes, *n* = 6 healthy kidney donors and *n* = 6 CKD patients). Rank of the processes is indicated on the y-axis (i.e. the higher the rank the more enriched). The adjusted *P*-value is indicated on the x-axis. The size of the dots indicates the number of genes involved within the specific GO term, shown as overlap ratio.

To link the transcriptome analysis to circulating calcification markers, calcification propensity (crystallization time, T_50_) and CPP counts in serum of healthy kidney donors and CKD patients were measured. Calcification propensity was significantly increased in CKD patients compared to healthy kidney donors (*Figure 5A*), as indicated by a lower T_50_ value (i.e. lower crystallization time of primary to secondary CPPs). This lower T_50_ in CKD coincided with an increased CPP2 diameter (*Figure 5B*). Numbers of both circulating CPP1 (primary CPPs, *Figure 5C*) and CPP2 (secondary CPPs, *Figure 5D*) were also significantly elevated in CKD compared to healthy kidney donors. Collectively, lower serum T_50_ and the resulting increase in numbers of circulating CPPs in CKD patients support the concept that CKD patients are at increased risk to develop (CPP-mediated) VC.

Subsequently, a simple regression analysis was performed using the 190 differentially expressed genes from the RNA sequencing database (as summarized in the Volcano plot shown in *Figure 4B*) to explore whether any of these genes were associated with calcification propensity (T_50_), CPP1, or CPP2 counts. Although no significant associations were observed with calcification propensity, a total of 67 genes were found to be significantly associated with CPP1 counts (see Supplementary material online, *Table S6*). Additionally, 51 genes were significantly associated with CPP2 counts (see Supplementary material online, *Table S6*). Gene enrichment analysis of either the 67 significantly associated genes for CPP1 or the 51 genes for CPP2 was performed with the Reactome2022 database (*Figure 5E* and *F*). For both the CPP1- and CPP2-associated genes, enrichment was observed in vascular remodelling-related pathways, including Extracellular Matrix Organization, Elastic Fibre Formation, Collagen Formation, Assembly of Collagen Fibrils and Other Multimeric Structures, and Collagen Biosynthesis and Modifying Enzymes. Differences between the CPP1 and CPP2 enrichment analyses (*Figure 5E* and *F*) can be found in ranking of the pathways (i.e. the higher the rank the more enriched) and the number of genes present within a certain pathway (i.e. overlap). To illustrate, for both the CPP1- or CPP2-associated genes, enrichment was found for the process Extracellular Matrix Organization. However, genes associated with process Elastic Fibre Formation were more present in the CPP1 associations (*Figure 5E*), and genes associated with the process Collagen Biosynthesis and Modifying Enzymes were more abundant in the CPP2 associations (*Figure 5F*). Taken together, these data indicate that both CPP1 and CPP2 counts are strongly associated with pathways involved in the process of vascular remodelling.

## 4. Discussion

The current study was designed to provide insight in the underlying processes involved in vascular calcification (VC) and dysfunction in CKD. Through bulk RNA sequencing on vascular biopsies from both healthy kidney donors and CKD patients, followed by gene enrichment analysis, a significant increase was demonstrated in processes, such as endothelial activation, inflammation, ECM remodelling, and ossification in CKD. Increased calcification scores, calcification propensity (lower T_50_), and increased circulating CPP counts and their size supported a pro-calcifying phenotype of CKD patients. When linking the presence of circulating pro-calcifying markers, to the vascular biopsy transcriptome, we identified a significant association between vascular remodelling processes and CPP1 and CPP2 counts. These findings indicate that CPPs may not only play a role in CKD-enhanced calcification, but also contribute to vascular remodelling in CKD, even in the absence of histology-proven calcification.

The analysis of the computed tomography (CT) data showed an increased presence of (macro)calcifications in renal and iliac arteries, as well as the total abdominal vasculature of CKD patients compared to the healthy kidney donors. Previous studies have established associations between decreased eGFR and higher urinary albumin–creatinine ratio (i.e. markers of renal dysfunction and glomerular kidney damage) with increased calcifications in the carotid arteries, coronary arteries, and aorta.^34–37^ We confirmed that in our cohort, loss of renal function was associated with an increased development of vascular (macro)calcifications. Interestingly, when assessing the presence of microcalcifications in the vascular biopsies of healthy kidney donors and CKD patients, only very limited numbers of biopsies with moderate to severe microcalcifications were observed without clear differences between healthy donors and CKD patients. This unexpected finding contrasts with the CT-findings, but could be explained by tissue sampling bias. During renal transplantation, the macroscopically most healthy and least or even non-calcified part of the iliac artery is typically selected to make the anastomosis with the graft renal artery. As a result, the collection of relative healthy vascular biopsies from CKD patients may bias towards a relatively healthy tissue, potentially underestimating the presence of microcalcifications in other parts of the vasculature. However, this does not exclude the possibility that molecular processes involved in VC or remodelling are already activated, even in the absence of histology-proven microcalcifications.

Therefore, to study molecular pathways underlying vascular dysfunction and VC in CKD, we performed bulk RNA sequencing, followed by gene enrichment analysis. For (cellular) biological processes ‘*smooth muscle cell differentiation, osteochondrogenic dedifferentiation and vascular remodelling*’, we observed enrichment in CKD biopsies compared to healthy kidney donors. The most clinically relevant observation from this enrichment analysis lies in the increased vascular remodelling observed in the CKD vascular tissue. Literature indicates a close relationship between vascular remodelling and development of VC.^38–40^ In response to various biological stressors, VSMC can alter their phenotype from contractile to a more synthetic phenotype, accompanied by increased VSMC proliferation, migration, and extracellular matrix (ECM) remodelling.^38^ Indeed, the CKD vascular biopsies showed enrichment for processes related to smooth muscle cell proliferation, migration, and ECM remodelling, including ECM assembly/disassembly and collagen catabolic processes, indicating a transition in the VSMC phenotype. During vascular remodelling, a key role can be attributed to matrix metalloproteases (MMPs), including MMP2 and MMP9, which gradually degrade the ECM.^41^ The ECM degradation can create newly formed niches for calcium deposition in the vascular wall as elastinolysis increases the affinity for calcium binding, thereby contributing to VC.^39^ Recent studies have shown that in arterial biopsies from CKD patients, upregulation of MMP2 and MMP9 was significantly associated with the incidence of medial calcification, arterial stiffness, and endothelial dysfunction.^42,43^ Interestingly, in the current study, we also found significant upregulation of MMP2 in the vascular tissue of CKD patients. Additionally, enrichment analysis of genes associated with CPP1 or CPP2 counts showed increased enrichment in vascular remodelling-related pathways, which strongly suggests that CPPs might influence the vascular remodelling process in CKD. Surprisingly, enriched pathways were quite similar among both CPP1- and CPP2-associated genes. Literature describes CPP2, and not CPP1, as the mature and toxic form of nanocrystals that affect the VSMC calcification process *in vitro*.^13,44^ In contrast, both particle types have inflammatory effects *in vitro*.^45,46^ Our data showed that both types of CPPs in humans *in vivo* are associated with vascular remodelling-related processes, indicating that although CPP2 are considered the marker for the VC process, the toxicity of CPP1 should not be underestimated, and both CPP1 and CPP2 might be involved in driving vascular pathology. One might ask whether increased CPP numbers cause altered gene expression profiles in the vascular wall or vice versa, where altered gene expression leads to increased CPP numbers. However, *in vitro* studies demonstrate direct effects of CPPs on endothelial cell activation and VSMC calcification.^13,44,47,48^ Moreover, clinical treatment with a phosphate binder results in decreased CPP numbers and reduced EC activation and VSMC calcification.^49^ Finally, using a miniature pig CKD model, Miura *et al.* recently provided experimental proof that removal of circulating CPPs using an alendronate adsorption column improved survival and alleviated complications, including vascular calcification and endothelial dysfunction.^50^ Taken together, these data make it highly plausible that CPPs are responsible for the observed differential gene expression, rather than vice versa.

In light of the current observations, the question arises as to whether distinct osteochondrogenic dedifferentiation of VSMC is actually present. Although some enrichment for osteoblast differentiation in the CKD patient biopsies was observed using RNAseq, no reduction was observed in the expression of the classical VSMC differentiation, such as ACTA2, MYH11, and TAGLN using qRT-PCR. Furthermore, no significant alterations in the expression of pro-calcifying VSMC markers like ALPL, IBSP, MSX2, RUNX2, and SOX9 were observed. Based on the combined RNAseq and qRT-PCR results, our data indicate that classical osteochondrogenic dedifferentiation may not be occurring in the vascular biopsies from CKD patients. The literature frequently describes osteochondrogenic dedifferentiation as fundamental process in VC. However, various studies have also shown that VC development can occur without VSMC osteochondrogenic dedifferentiation. For example, a recent *in vitro* study showed that, in response to high phosphate levels (2 mM Pi), VSMC started to calcify both in living and fixed conditions,^51^ indicating that passive calcification can occur in the absence of active cellular processes in VSMC. Similarly, a study investigating the beneficial effect of magnesium (Mg^2+^) supplementation on VC did not observe alterations in classical dedifferentiation markers despite decreased VSMC calcium deposition.^52^ While VSMC are frequently considered to transform to osteoblast-like cells, the expression of osteoblast-like genes in VSMC in calcified areas remains relatively low.^53^ Whether or not VSMC osteochondrogenic dedifferentiation is a prerequisite for VC, using qRT-PCR, we did not observe differential expression of the classical dedifferentiation markers in the collected vascular biopsies, although RNAseq revealed the cellular process ‘ossification’ to be significantly enriched in the CKD biopsies compared with kidney donors. The parallels between VC and bone formation and mineralisation are noteworthy.^54^ During bone formation, the collagen-rich ECM is first created, followed by the onset of the mineralisation process. Based on our results, we suggest that in VC, remodelling of the vasculature, including ECM reorganisation, rather than VSMC osteochondrogenic dedifferentiation, provides a new environment, which is more prone to calcify and therefore contributes to the development of VC.

A remarkable observation from the enrichment analysis is the increased endothelial activation, inflammation, nitric oxide (NO) signalling, and cellular redox profiles in CKD biopsies. We recently showed that CPPs activate ECs, impair the NO signalling, and elevate superoxide (O_2_^−^) production *in vitro*.^47^ Also, *in vitro* and animal studies have shown that CPPs increase endothelial cytokine production, chemokine secretions, and circulating levels of soluble ICAM-1 and IL-8.^46,55^ As already described above, a causal role of CPPs in the development of VC and endothelial dysfunction was supported by a recent study indicating that removal of CPPs from the circulation in miniature pigs improves VC, endothelial function, chronic inflammation, and mortality.^50^ Furthermore, in a recent study with haemodialysis patients, it was shown that removing CPP from sera with a non-calcium-based phosphate binder ameliorated the propensity of patient serum to activate ECs *in vitro*.^49^ Combining these findings with our data leads us to propose that the presence of increased circulating CPP counts in CKD may lead to EC activation and dysfunction, contributing to vascular dysfunction, remodelling, and VC. Whether CPP are causally involved in development of endothelial dysfunction and VC in human CKD remains to be established.

A strength of the current study is the comprehensive vascular tissue analysis from both healthy kidney donors and CKD patients. Whereas most studies investigate the association between CPPs, calcification propensity, and less invasive parameters, such as serum inflammatory markers, eGFR, arterial stiffness (i.e. pulse-wave velocity), disease prevalence or mortality rate, we were able to collect unique vascular tissue biopsies. This allowed for a thorough transcriptome analysis, enabling us to explore not only calcification markers, but also processes involved in vascular remodelling, such as endothelial activation, endothelial to mesenchymal phenotype transition, and endothelial senescence.

Since obtaining human vascular tissue is challenging due to ethical constraints, this makes the current study valuable and provides additional insight in the processes involved in VC and vascular remodelling in CKD. One limitation of the study is the possible effect of dialysis on the vascular tissue. It has been shown that (haemo)dialysis might cause an inflammatory response.^56^ Whether this also affects the vascular tissue of the CKD patients cannot be excluded. Additionally, the source of the vascular tissue between healthy kidney donors (renal artery) and CKD patients (external iliac artery) differs. Although these arteries exhibit great similarity in size and other morphological features,^57^ potential intrinsic differences on the molecular level cannot be excluded. Yet, when contrasted against the gene expression profile of CKD renal arteries, the small difference in differential gene expression profiling (0.70%) between the MOD iliac arteries and the KD renal arteries suggests that CKD has a more pronounced effect on gene expression changes than the anatomical site.

Another possible limitation is the relatively small number of samples used for the RNAseq analysis. The data presented in this manuscript should primarily be viewed as a starting point for future comparable studies, contributing to the generation of new hypotheses regarding the role of CPPs in the development of vascular calcifications. Lastly, gene expression profiles derived from RNA-sequencing experiments may be confounded by variation due to technical artefacts, including sequencing batch, RNA integrity, sampling location, and processing time. Such confounding factors are commonly mitigated by multivariate adjustment methodologies to regress out the unmeasured confounding variables.^58,59^ Although in our study, we made every effort to limit the effects of such confounding factors, the limited availability of clinical samples makes multivariate adjustment inappropriate.

In conclusion, our in-depth transcriptome profiling of both CKD and healthy kidney donor vascular tissue, combined with the analysis of CPP counts and calcification propensity, reveals a significant association between CPP counts and markers of vascular remodelling, potentially predisposing individuals for VC development. Interventions targeting processes involved in CPP-induced vascular remodelling and VC emerge as promising targets to mitigate enhanced vascular disease in CKD.

**Translational perspective**

Vascular calcification (VC) is frequently observed in chronic kidney disease (CKD) and contributes to cardiovascular morbidity and mortality. Current research focuses on linking circulating pro-calcifying determinants (calciprotein particles (CPPs), calcification propensity) with disease progression. The understanding of the underlying processes of VC and vascular dysfunction in CKD is limited. Using transcriptome analysis on the vascular tissue from healthy donors and CKD patients, gene enrichment in processes like endothelial activation, inflammation, ECM remodelling, and ossification was demonstrated in CKD biopsies, with remodelling markers being significantly associated with CPP counts. Interventions affecting CPPs are promising therapeutic avenues for alleviating vascular dysfunction (i.e. vascular remodelling and VC) in CKD.

## Supplementary Material

cvae164_Supplementary_Data

## Data Availability

All data are incorporated into the article and its online Supplementary material. Raw data of RNA sequencing are available as Gene Expression Omnibus (GEO) DataSet (pending at the time of publication).
