# Supplementary material for: Calciprotein particle counts associate with vascular remodelling in chronic kidney disease
Source: Cardiovasc Res. 2024 Aug 5;120(15):1953–66. doi: 10.1093/cvr/cvae164 (PMC11629976; doi:10.1093/cvr/cvae164)
Supplement: cvae164_Supplementary_Data [file cvae164_supplementary_data.zip › Supplementary Materials_CVR-2024-0182R_final_August2024.docx]

**----------------------------------------------- SUPPLEMENTARY MATERIALS --------------------------------------------**

**CALCIPROTEIN PATICLE COUNTS ASSOCIATE WITH VASCULAR REMODELLING IN CHRONIC KIDNEY DISEASE**

**SHORT TITLE:** Calciprotein particles and vascular remodelling in CKD

Lian Feenstra^1*^, Melanie Reijrink^1,2*^, Andreas Pasch^3,4^, Edward R. Smith^5,6^, Lotte M. Visser^1^, Marian Bulthuis^1^, Monique E. Lodewijk^1^, Mirjam F. Mastik^1^, Marcel J.W. Greuter^7^, Riemer H.J.A. Slart^8,9^, Douwe J. Mulder^2^, Robert A. Pol^10^, Charlotte A. te Velde-Keyzer^11^, , Guido Krenning^13†^, Jan-Luuk Hillebrands^1†**^, TransplantLines Investigators^12^

^1^Department of Pathology and Medical Biology, University of Groningen, University Medical Center Groningen, Groningen, the Netherlands

^2^Department of Internal Medicine, Division of Vascular Medicine, University of Groningen, University Medical Center Groningen, Groningen, the Netherlands

^3^Calciscon AG, Biel, Switzerland

^4^Institute of Physiology and Pathophysiology, Johannes Kepler University Linz, Linz, Austria

^5^Department of Nephrology, Royal Melbourne Hospital, Parkville, Victoria, Australia

^6^Department of Medicine, University of Melbourne, Parkville, Victoria, Australia
^7^Department of Radiology, Medical Imaging Center, University of Groningen, University Medical Center Groningen, Groningen, the Netherlands
^8^Department of Nuclear Medicine and Molecular Imaging, Medical Imaging Center, University of Groningen, University Medical Center Groningen, Groningen, the Netherlands
^9^Department of Biomedical Photonic Imaging, Faculty of Science and Technology, University of Twente, Enschede, the Netherlands
^10^Department of Vascular and Transplant Surgery, University of Groningen, University Medical Center Groningen, Groningen, the Netherlands

^11^Department of Internal Medicine, Division of Nephrology, University of Groningen, University Medical Center Groningen, Groningen, The Netherlands

^12^TransplantLines Investigators: Adelita V. Ranchor, Antonio W. Gomes Neto, Arjan Diepstra, Bouke G. Hepkema, C. Tji Gan, Caecilia S.E. Doorenbos, Charlotte A. te Velde-Keyzer, Coretta van Leer-Buter, Daan J. Touw, Eelko Hak, Erik A.M. Verschuuren, Frank A.J.A. Bodewes, Frank Klont, Gerard Dijkstra, Gertrude J. Nieuwenhuis-Moeke, Hans Blokzijl, Henri G.D. Leuvenink, Hubert G.M. Niesters, J. Cas Swarte, Jan-Stephan F. Sanders, Kevin Damman, L. Joost van Pelt, Marco van Londen, Marieke T. de Boer, Marion J. Siebelink, Marius C. van den Heuvel, Michel J. Vos, Michiel E. Erasmus, Rianne M. Douwes, Riemer J.H.J.A. Slart, Rinse K. Weersma, Robert A. Pol, Robert J. Porte, Vincent E. de Meijer, Willem S. Lexmond

^13^Department of Clinical Pharmacy and Pharmacology, Division Experimental Pharmacology, University of Groningen, University Medical Center Groningen, Groningen, The Netherlands

**^*,^**^†^ **AUTHORS CONTRIBUTED EQUALLY**

**^**^ CORRESPONDING AUTHOR:**

J.L. Hillebrands, PhD

Professor of Experimental Vascular Pathology
University Medical Center Groningen, Department of Pathology and Medical Biology
Hanzeplein 1, 9713 GZ Groningen, The Netherlands
E-mail address: [j.l.hillebrands@umcg.nl](mailto:j.l.hillebrands@umcg.nl)

Phone: +31 (0)6 25651329

**Supplemental Table S1: Primer sequences used for RT-qPCR.**

| Target | Primer sequence forward | Primer sequence reverse | Accession number and UniGene assay ID (TaqMan) | Amplicon size (bp) |
| --- | --- | --- | --- | --- |
| β-actin | CCTGGCACCCAGCACAAT | CCGATCCACACGGAGTAC | NM_001101.5 | 69 |
| ACTA2 | GCCAAGCACTGTCAGGAATC | TTGTCACACACCAAGGCAGT | NM_001613.3 | 80 |
| MYH11 | CCGGGAAAACCGAAAACACC | CCAGCTCTCCGTAGGCAAAA | NM_001040113.1 | 120 |
| TAGLN | GCAGCCCTTTAAACCCCTCA | CATGTCTGGGGAAAGAAGGCT | NM_003186.4 | 113 |
| ALPL | CTATCCTGGCTCCGTGCTC | TTAACTGATGTTCCAATCCTGCG | NM_000478.5 | 82 |
| IBSP | ATTTTGGGAATGGCCTGTGC | GGCCTGTACTTAAAGACCCCA | NM_004967.3 | 95 |
| MGP | GTCCAAGAGAGGATCCGAGAAC | AGCGTTCGCAAAGTCTGTAGT | NM_000900.4 | 85 |
| MSX2 | GCCTCGGTCAAGTCGGAAAA | AGGGCTCATATGTCTTGGCG | NM_002449 | 90 |
| RUNX2 | AGATTTGTGGGCCGGAGTG | GTCTGTGCCTTCTGGGTTCC | NM_001278478.1 | 139 |
| SOX9 | GCTCTGGAGACTTCTGAACGA | CCGTTCTTCACCGACTTCCT | NM_000346 | 132 |

β-actin= beta actin, ACTA2= actin alpha 2, MYH11= myosin heavy chain 11, ALPL= alkaline phosphatase, IBSP= integrin binding sialoprotein, MGP= matrix Gla protein, MSX2 = muscle segment homeobox 2, RUNX2= runt-related transcription factor 2, SOX9= SRY-Box transcription factor.

**Supplemental Table S2: Baseline characteristics of chronic kidney disease (CKD) patients, healthy kidney donors, and multi-organ donors (MOD) used for artery biopsy RNAseq analysis.**

|  | **Chronic kidney disease patients (CKD, N=6)** | **Healthy kidney**  **donors (N=6)** | **Multi-organ**  **donors (MOD, N=6)** | **p-value** |
| --- | --- | --- | --- | --- |
| **Male gender (%)** | 50 | 33.3 | 50 | 0.799 |
| **Age (years)** | 54 [31 - 61] | 54 [39 - 66] | 55 [49 -64] | 0.754 |
| **Body mass index (kg/m^2^)** | 27 ± 3.9 | 27 ± 3.3 | 27 ± 2.3 | 0.964 |
| **Dialysis (%)** | 100 | 0 | 0 | 0.055 |
| **Estimated glomerular filtration rate (mL/min/1.73m^2^)** | 6.0 [5.3 -8.5] | 93 [67 - 109] |  | 0.002 |
| **Type 2 diabetes (%)** | 16.7 | 0 | 0 | 0.347 |
| **Diabetes duration (years)** | 8.00 |  |  |  |
| **HbA1_C_ (%)** | 5.4 ± 0.7 | 5.4 ± 0.1 | 5.4 ± 0.2^*^ | 0.995 |
| **Total cholesterol (mmol/L)** | 4.7 ± 1.3 | 4.5 ± 0.6 |  | 0.745^**^ |
| **C-reactive protein (mg/L)** | 4.3 [1.7 - 9.8] | 1.0 [0.3 - 15.7] | 49.0 [12.3 - 96.3] | 0.019 |
| **Leukocyte count (10^9^/L)** | 7.8 [4.7 - 8.9] | 6.2 [5.6 - 6.9]**^‡^** | 9.3 [7.5 - 24.1] | 0.072 |
| **Calcium (mmol/L)** | 2.4 ± 0.1 | 2.3 ± 0.1 | 1.9 ± 0.5**^‡^** | 0.032 |
| **Phosphate (mmol/L)** | 1.7 ± 0.3 | 0.9 ± 0.2 |  | 0.002^**^ |
| **Alanine aminotransferase (U/L)** | 23.5 [13.5 - 30.3] | 23.0 [17.5 - 33.3] | 29.5 [22.0 -76.3] | 0.364 |

Data are presented as mean ± SD (when normally distributed) or as median with [IQR] (when not normally distributed). ^*^N=4, ^‡^N=5, p-value based on One-Way ANOVA test when normally distributed (^**^or unpaired t-test), or Kruskal-Wallis test when not-normally distributed, or Chi-squared test for categorical data (gender, dialysis and Type 2 diabetes mellitus). Some MOD data were missing since respective data were not included in donor reports from external procurement centers.

| Supplemental Table S3: Comparison between qPCR and RNAseq data for VSMC dedifferentiation and calcification genes. | | | | | | | | | | | | |
| --- | --- | --- | --- | --- | --- | --- | --- | --- | --- | --- | --- | --- |
|  | | **qPCR data** | | |  |  | | | **RNAseq data** | | | |
|  | **Log_2_ FD** | | **S.E.M.** | **n** | | |  | **Log_2_ FD** | | **S.E.M.** | **n** | **p-value** |
| ACTA2  MYH11  TAGLN  ALPL  IBSP  MGP  MSX2  RUNX2  SOX9 | 0.487  0.000  0.002  0.000  0.000  0.796  0.597  0.000  0.001 | | 0.111  0.000  0.002  0.000  0.000  0.167  0.040  0.000  0.000 | 33  33  33  33  33  33  33  33  33 | | |  | -0.124  -0.115  -0.212  0.09  n.d.  0.438  n.d.  0.369  0.36 | | 0.329  0.277  0.278  0.455  0.376  0.432  0.439 | 6  6  6  6  6  6  6 | >0.999  >0.999  >0.999  >0.999  >0.999  >0.999  >0.999 |
| Log_2_ FD: Log_2_ fold difference in expression between CKD patients and living kidney donors; n.d.: not detected | | | | | | | | | | | | |

***
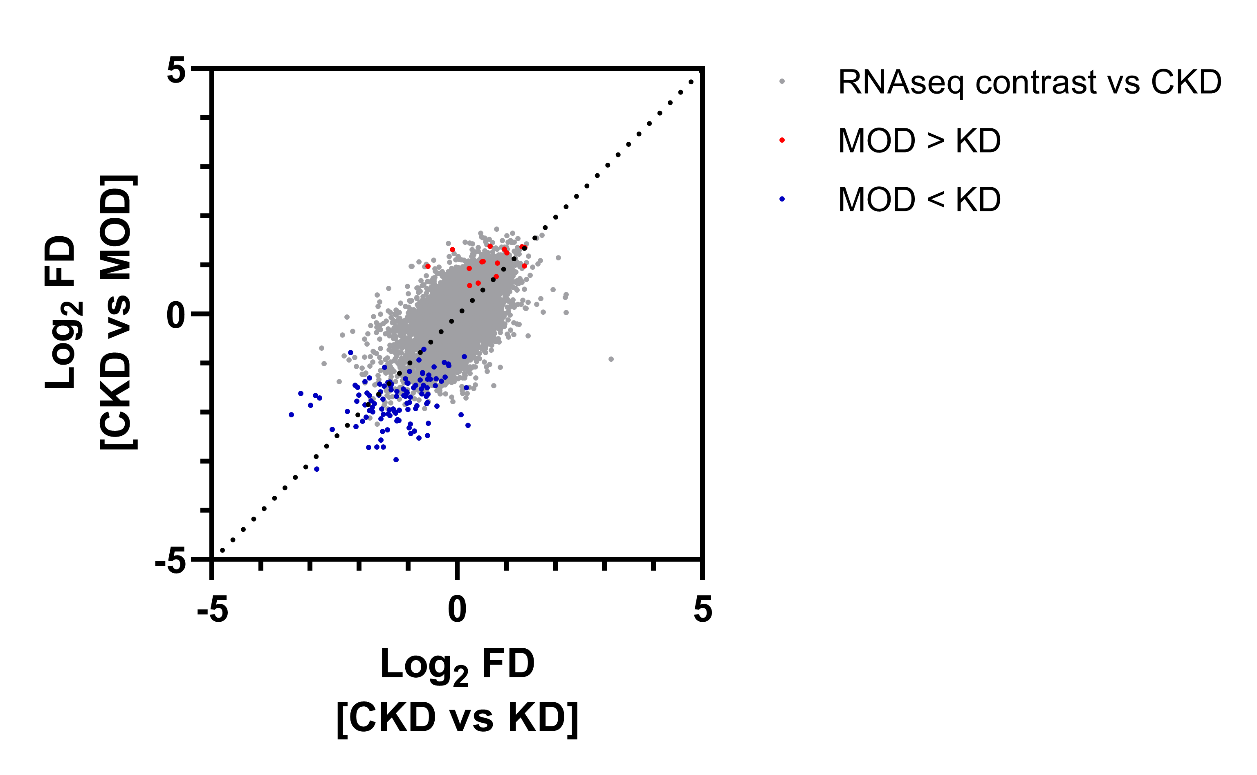
***

**Supplemental Figure 1: Comparison of Log_2_ fold difference (Log_2_FD) of ‘CKD vs MOD’ with ‘CKD vs KD’.** Differential expression of 15 genes (red dots) was higher and of 112 genes (blue dots) was lower in MOD external iliac arteries compared with KD renal arteries.
